# Trends and Healthcare Profiles of Older Asian American Adults With ADRD in U.S. Nursing Homes

**DOI:** 10.1093/geroni/igaf122.3192

**Published:** 2025-12-31

**Authors:** Yiyang Yuan, Bei Wu, Kate Lapane

**Affiliations:** University of Massachusetts Chan Medical School, Worcester, Massachusetts, United States; NYU Shanghai, China (People’s Republic); University of Massachusetts Chan Medical School, Worcester, Massachusetts, United States

## Abstract

The growing population of older Asian American adults with ADRD, increasing demand for nursing homes, and prevalent preference for non-English languages, which may contribute to social isolation, underscore the need to examine trends and healthcare profiles of non-Hispanic Asian adults with ADRD in U.S. nursing homes. Using Minimum Data Set 3.0, we identified 55,693 non-Hispanic Asian older adults newly admitted for skilled nursing facility (SNF) care and 59,063 for non-SNF care. We report key findings for SNF residents, as results were largely consistent. From 2011 to 2022, the proportion of older non-Hispanic Asian residents with ADRD doubled from 1.2% to 2.4%, with the highest annual growth rate (0.09%, p < 0.0001) among all racial/ethnic groups. About 40% required an interpreter. The top preferred non-English languages were Chinese (17.4%), Korean (12.8%), and Vietnamese (8.1%). Average ages ranged from 82.8 (SD: 7.9, Vietnamese-preferred) to 85.4 years (SD: 7.4, Chinese-preferred). Compared to non-Hispanic White, English-preferred residents, non-Hispanic Asian residents had higher rates of admission from acute hospitals, higher prevalence of severe cognitive impairment, frailty, hypertension, diabetes, and stroke, and higher CHESS and mortality risk scores. Notable within-group variations included frailty proportions from 66.9% (Korean-preferred) to 81.4% (Chinese-preferred), severe cognitive impairment from 12.5% (English-preferred) to 19.3% (Vietnamese-preferred), and average mortality risk scores from 5.2 (SD: 2.6, Korean-preferred) to 6.2 (SD: 3.0, Chinese-preferred). The growing presence and diverse healthcare needs of non-Hispanic Asian residents with ADRD emphasize the necessity of understanding health outcomes by specific subgroups and culturally and linguistically tailored care to address specific challenges.

